# Ureteral triplication with a contralateral duplication and ureterocele: a case report

**DOI:** 10.4076/1757-1626-2-7510

**Published:** 2009-06-26

**Authors:** Fayza Alhajri, Ammar Al-Jumah, Sara Al-Mutawa

**Affiliations:** Department of Clinical Radiology, Mubarak Al-Kabeer HospitalJaberiyaKuwait

## Abstract

We report a 10-year-old boy who presented with nocturnal enuresis. Radiological workup revealed a left ureteral triplication (Smith type 2) with a contralateral duplication and ureterocele. This presentation and its association are extremely rare. The clinical and radiological features are presented here as early diagnosis is important to avoid complications and future renal damage.

## Introduction

Ureteral triplication is a rare congenital anomaly of the upper urinary tract. We present a 10-year old child with a triplication of ureter on the left side and a ureteral duplication with an associated ureterocele on the right side. Due to lack of specific clinical signs, the mainstay of diagnosis remains a radiological one which can direct the best treatment strategy.

## Case presentation

A 10 year old Arabian boy from Egypt presented with nocturnal enuresis which was 2-3 times a week. There was no history of flank pain or recurrent urinary tract infections. The clinical examination of the patient was normal with normal developmental assessment. Urine analysis and culture were normal. Routine blood tests including full blood count, serum urea and electrolytes were normal. There was no family history of relevance.

**Figure 1.**
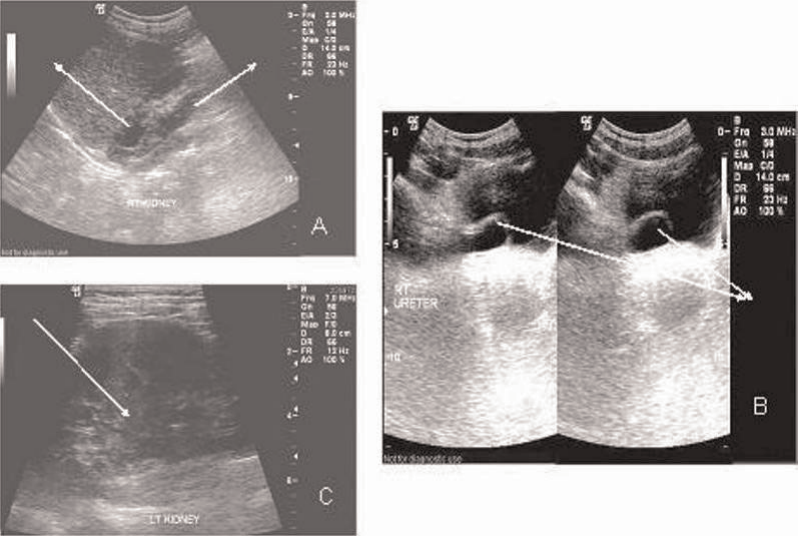
**(A)** Ultrasound examination of the right kidney showing the duplex collecting system (arrow) with moderate dilatation of the upper moiety. **(B)** Ultrasound examination of the urinary bladder showing the right sided ureterocele (arrow) at the ureterovesical junction. **(C)** Ultrasound examination of the left kidney showing the central parenchyma separating the kidney moieties (arrow).

The patient underwent ultrasound examination of the kidneys that showed evidence of right duplex kidney with dilatation of the calyces of the upper moiety (Figure 1A). The right upper moiety ureter was moderately dilated along its length and ends into ureterocele in the right side of bladder base. The ureterocele was noted as intravesical thin walled cystic dilatation at the distal end of the ureter (Figure 1B).The left kidney shows apparent central parenchyma separating two moieties with no evidence of obstruction noted (Figure 1C).

**Figure 2.**
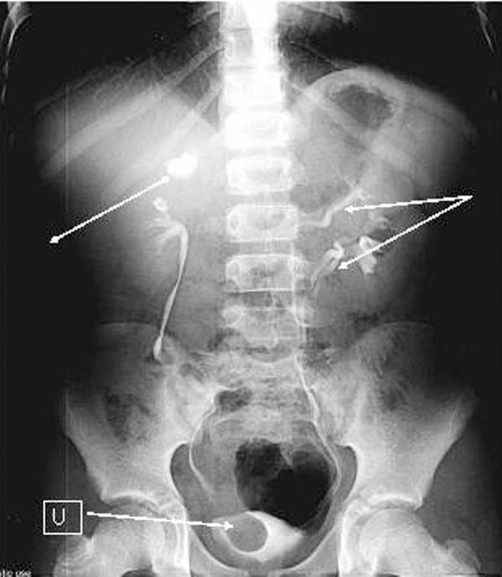
Intravenous urography (15 minutes film) showing left ureteral triplication (single head arrow) with fusion of two ureters at the level of first sacral vertebra. Contralateral duplication with hydronephrosis (double arrow) of the upper moiety and the ureterocele (U) is noted.

**Figure 3.**
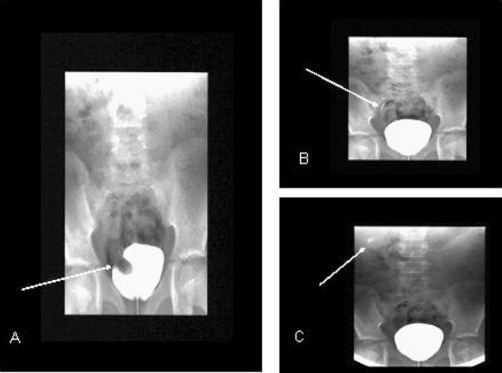
MCUG examination showing right sided ureterocele (**A**, arrow), grade 1 VUR in the right ureter (**B**, arrow) and lower pole moiety of the right kidney (**C**, arrow).

Intravenous urography (IVU) (Figure 2) showed right duplex kidney and duplex ureter with hydronephrosis of upper pole moiety and an associated ureterocele appearing as a filling defect in the right bladder base. There was triplication of the ureter on the left side with two ureters joining before entering the bladder (Smith’s type 2). Micturating cystourethrogram (MCUG) showed grade 1 vesicoureteric reflux (VUR) in the lower pole moiety ureter of the right kidney (Figure 3). The patient underwent cystoscopic examination which showed a large ureterocele on the right side that was punctured to relieve the obstruction. Cystoscopy also confirmed the presence of two ureteric orifices on each side. Patient was discharged home two days later with no complications and is currently under surveillance. Family members were also screened by ultrasound examination which was normal.

## Discussion

Ureteral duplication occurs in 0.3-0.8% of the population, but ureteral triplication has been very rarely described [1]. Ureteral triplication is a developmental abnormality of the ureteral bud originating from the Wolffian duct at the 5^th^ week of embryological life. The ureteral bud arises from the distal part of the wollfian duct after 4 weeks of foetal development [2]. It grows dorsally at first and cranially later and makes contact with the metanephros and the distal end differentiates into the renal pelvis and the major and minor calyces during the 6^th^-8^th^ weeks. In triplication of the ureter, three ureteral buds could arise independently from the mesonephric duct or from early fission of one or more ureteral buds to join the metanephros [2]. Smith [3] classified ureteral triplication into 4 types as follows: Complete ureteral triplication (35%), incomplete triplication (21%), trifid ureters (31%), double ureter, and one bifurcated (9%).

Our case was Smith type 2 triplication. Triplication is more common in females and on the left side [2]. Presenting symptoms are renal colic, recurrent UTI, and urinary incontinence [2,4]. In addition, patients with triplication may encounter symptoms and signs of reflux, obstruction, ureterocele, or ectopia, similar to those with duplication anomalies [1]. The most frequently encountered urological anomalies associated with ureteral triplication are: contralateral duplication (37%), ureteral ectopia (28%), renal dysplasia (8%) and reflux [2]. Treatment is best tailored to individual cases and depends on the extent of the clinical problem i.e. presence of obstruction, extravesical ureteral ectopia, ureterocele or VUR. Transurethral incision of ureterocele effectively relieves the obstruction but may result in VUR necessitating ureteral re-implantation at a later stage. Other forms of surgical intervention require partial nephrectomy with aspiration of ureterocele in the first instance with re-implantation of ureter at a later stage [5].

Ureteral triplication is a rare congenital anomaly that requires high index of suspicion in order to diagnose. Due to lack of specific clinical signs, radiological examination remains the mainstay of diagnosis and can direct the best treatment strategy. This case emphasizes the importance of complete anatomic and functional evaluation of the urinary tract in the management of a child with urinary symptoms.

## Abbreviations

IVU: intravenous urography
MCUG: micturating cystourethrogram
VUR: vesicoureteric reflux
